# Supplementary material for: Sensitive detection of SARS-CoV-2 seroconversion by flow cytometry reveals the presence of nucleoprotein-reactive antibodies in unexposed individuals
Source: Commun Biol. 2021 Apr 20;4:486. doi: 10.1038/s42003-021-02011-6 (PMC8058339; doi:10.1038/s42003-021-02011-6)
Supplement: Supplementary file 3 — Description of Supplementary Files [file 42003_2021_2011_MOESM3_ESM.pdf]

Description of Additional Supplementary Files

File name: Supplementary Data 1

Description: Source data corresponding to gMFI and OD values underlying plots shown in figures.
