# Supplementary material for: Sensitive detection of SARS-CoV-2 seroconversion by flow cytometry reveals the presence of nucleoprotein-reactive antibodies in unexposed individuals
Source: Commun Biol. 2021 Apr 20;4:486. doi: 10.1038/s42003-021-02011-6 (PMC8058339; doi:10.1038/s42003-021-02011-6)
Supplement: Supplementary file 5 — Reporting Summary [file 42003_2021_2011_MOESM5_ESM.pdf]

nature research

Corresponding author(s): Asis Palazon

Last updated by author(s): Mar 5, 2021

Reporting Summary

Nature Research wishes to improve the reproducibility of the work that we publish. This form provides structure for consistency and transparency in reporting. For further information on Nature Research policies, see our Editorial Policies and the Editorial Policy Checklist.

Statistics

For all statistical analyses, confirm that the following items are present in the figure legend, table legend, main text, or Methods section.

n/a	Confirmed	
☐	☒	The exact sample size (*n*) for each experimental group/condition, given as a discrete number and unit of measurement
☐	☒	A statement on whether measurements were taken from distinct samples or whether the same sample was measured repeatedly
☐	☒	The statistical test(s) used AND whether they are one- or two-sided<br>*Only common tests should be described solely by name; describe more complex techniques in the Methods section.*
☐	☒	A description of all covariates tested
☒	☐	A description of any assumptions or corrections, such as tests of normality and adjustment for multiple comparisons
☐	☒	A full description of the statistical parameters including central tendency (e.g. means) or other basic estimates (e.g. regression coefficient) AND variation (e.g. standard deviation) or associated estimates of uncertainty (e.g. confidence intervals)
☐	☒	For null hypothesis testing, the test statistic (e.g. F, t, r) with confidence intervals, effect sizes, degrees of freedom and P value noted<br>*Give P values as exact values whenever suitable.*
☒	☐	For Bayesian analysis, information on the choice of priors and Markov chain Monte Carlo settings
☒	☐	For hierarchical and complex designs, identification of the appropriate level for tests and full reporting of outcomes
☒	☐	Estimates of effect sizes (e.g. Cohen's *d*, Pearson's *r*), indicating how they were calculated

Our web collection on statistics for biologists contains articles on many of the points above.

Software and code

Policy information about availability of computer code

Data collection	Data was collected using FACSDiva software (BD Biosciences).
Data analysis	Flow cytometry results were analyzed using FlowJo version 10 (BD Biosciences). Statistical analyses were performed using Prism version 8 (GraphPad). Phylogram generated from the FASTA alignment file was performed using FastTree (https://www.genome.jp/). Sensitivity and specificity values (ROC curves) were calculated by R

For manuscripts utilizing custom algorithms or software that are central to the research but not yet described in published literature, software must be made available to editors and reviewers. We strongly encourage code deposition in a community repository (e.g. GitHub). See the Nature Research guidelines for submitting code & software for further information.

Data

Policy information about availability of data

All manuscripts must include a data availability statement. This statement should provide the following information, where applicable:

- Accession codes, unique identifiers, or web links for publicly available datasets
- A list of figures that have associated raw data
- A description of any restrictions on data availability

All data generated or analysed during this study are included in this published article (and its supplementary information files).

Field-specific reporting

Please select the one below that is the best fit for your research. If you are not sure, read the appropriate sections before making your selection.

[x] Life sciences   [ ] Behavioural & social sciences   [ ] Ecological, evolutionary & environmental sciences

For a reference copy of the document with all sections, see nature.com/documents/nr-reporting-summary-flat.pdf

Life sciences study design

All studies must disclose on these points even when the disclosure is negative.

Sample size	Sample size was determined/limited by availability of samples.
Data exclusions	No data were excluded from the analysis.
Replication	Assays were repeated at least two times each.
Randomization	Randomization was not performed since the purpose of this work was assay development.
Blinding	Blinding was not performed since the purpose of this work was assay development.

Reporting for specific materials, systems and methods

We require information from authors about some types of materials, experimental systems and methods used in many studies. Here, indicate whether each material, system or method listed is relevant to your study. If you are not sure if a list item applies to your research, read the appropriate section before selecting a response.

Materials & experimental systems

n/a	Involved in the study	
[ ]	[x]	Antibodies
[x]	[ ]	Eukaryotic cell lines
[x]	[ ]	Palaeontology and archaeology
[x]	[ ]	Animals and other organisms
[ ]	[x]	Human research participants
[x]	[ ]	Clinical data
[x]	[ ]	Dual use research of concern

Methods

n/a	Involved in the study	
[x]	[ ]	ChIP-seq
[ ]	[x]	Flow cytometry
[x]	[ ]	MRI-based neuroimaging

Antibodies

Antibodies used

Biotinylated human IgG (Novus Biologicals #NBP1-96855), Biotinylated human IgM (Novus Biologicals #NBP1-96989), Commercial antibodies against RBD (GenScript #A02038) and N (Acrobiosystems #NUN-S41), Anti-human IgG-PE (BD Biosciences #555787) and IgM-BV421 (BD Biosciences #555783), Anti-human IgG-horseradish peroxidase (HRP) conjugated secondary antibody (GenScript #A01854) and Anti-human IgM-HRP (Novus Biologicals #NBP1-75014).

Validation

Novus Biologicals #NBP1-96855
Manufacter's statement: Human IgG whole molecule Biotin conjugated was assayed by immunoelectrophoresis resulted in a single precipitin arc against anti-biotin, antiHuman IgG and anti-Human Serum.
Publication: doi: 10.7150/thno.27221; PMID: 30429890.

Novus Biologicals #NBP1-96989
Manufacter's statement: Assay by immunoelectrophoresis resulted in a single precipitin arc against anti-biotin, anti-Human IgM and anti-Human Serum. No reaction was observed against anti-Human IgG F(c) or anti-Pepsin.

GenScript #A02038
Manufacter's statement: The product is specific for SARS-CoV-2 Spike Protein S1 subunit and its RBD domain.

Acrobiosystems #NUN-S41
Manufacter's statement: The cross-reactivity with other coronaviruses has not been tested yet.

BD Biosciences #555787
Manufacter's statement: The G18-145 monoclonal antibody specifically binds to the heavy chain of human immunoglobulin G subclasses: IgG1, IgG2, IgG3 and IgG4. The G18-145 antibody has been reported not to react with the heavy chains of other human immunoglobulin isotypes.

Publication: PMID 1723668.

BD Biosciences #555783
Manufacter's statement: The G20-127 monoclonal antibody binds to the heavy chain of human IgM. The G20-127 antibody is not thought to react with other immunoglobulin heavy chain isotypes.
Publication: PMID 1723668.

GenScript #A01854
Manufacter's statement: GenScript Mouse Anti-Human IgG Fc Antibody (50B4A9)[HRP], mAb reacts with the Fc portion of human IgG but not with the Fab portion of human IgG. This monoclonal antibody has no cross-reactivity with mouse, rat, rabbit, chicken or goat immunoglobulins.

Novus Biologicals #NBP1-75014
Manufacter's statement: Based on IEP, this antibody reacts with heavy mu chains on human IgM. Based on IEP, no reactivity is observed to non-immunoglobulin human serum proteins and light chains on all human immunoglobulins
Publication: doi: 10.1093/rheumatology/ker010; PMID: 21454308.

Human research participants

Policy information about studies involving human research participants

Population characteristics
The COVID cohort corresponds to 43 patients presenting COVID-19 symptomatology and diagnosed by PCR. The preCOVID cohort (50 serum samples) was obtained during the yearly medical check-up of the working population of the Basque Country in 2018-2019. Additional serum samples from negative controls (n=18) and independent COVID-19 cohorts confirmed by PCR were obtained after written informed consent and approval by the Cantabria Ethics Committee (CEIm Code: 2020.167). Serum samples from 34 patients with active infection were obtained at time of hospital admission, and samples from 20 convalescent patients were obtained one month after recovery from COVID-19.

Recruitment
The Basque Biobank managed sample storage and distribution from recruiting Hospitals.

Ethics oversight
93 samples corresponding to pre-Covid and acute Covid cohorts were provided by the Basque Biobank (www.biobancovasco.org) after approval from the corresponding ethics committee (CEIC-E 20-26, 1-2016). Additional serum samples from negative controls and independent COVID-19 cohorts confirmed by PCR were obtained after written informed consent and approval by the Cantabria Ethics Committee (CEIm Code: 2020.167).

Note that full information on the approval of the study protocol must also be provided in the manuscript.

Flow Cytometry

Plots

Confirm that:

- [x] The axis labels state the marker and fluorochrome used (e.g. CD4-FITC).
- [x] The axis scales are clearly visible. Include numbers along axes only for bottom left plot of group (a 'group' is an analysis of identical markers).
- [x] All plots are contour plots with outliers or pseudocolor plots.
- [x] A numerical value for number of cells or percentage (with statistics) is provided.

Methodology

Sample preparation
Microbead coating. PMMA (polymethyl methacrylate) 8.2 µm microbeads coated with streptavidin were purchased from PolyAn (Cat#10652009). Each microbeads presented a different fluorescence intensity (Red4 dye, Excitation: 590-680 nm/ Emission: 660-780 nm). First, microbeads were washed with cold PBS pH 7.2 (Gibco Cat#14190-094) by centrifugation at 2000 rpm for 5 min and resuspended in PBS. Then, biotinylated recombinant RBD, S1 and N (Acrobiosystems Cat#SPD-C82E9, Cat#S1N-C82E8 and Cat#NUN-C81Q6, respectively) were added to the tubes (RBD at 11 µg/mL, S1 at 30 µg/mL, N at 19,5 µg/mL) and kept at 4°C on rotating head over tail for an hour, protected from light. Positive control beads were coated with biotinylated human IgG (Novus Biologicals Cat#NBP1-96855) and IgM (Novus Biologicals Cat#NBP1-96989) on the same microbead at 15 µg/mL each. Negative control beads were not coated with protein. After the coupling reaction, microbeads were washed three times with PBS. Then, D-biotin (2 µM) (Sigma-Aldrich Cat#8512090001) was added and incubated for 15 min at room temperature (RT) to inactivate residual streptavidin. After three additional washes, equal amounts of each microbead were combined in the same vial.

C19BA assay. Antigen-coupled microbeads were added to protein LoBind 1.5 mL Eppendorf tubes (Eppendorf Cat#525-0133) in a volume of 50 µL of PBS containing a total of 5000-6000 beads. After centrifugation (2000 rpm, 5 min), microbeads were resuspended with 100 µL of pre-diluted (PBS) serum samples or serially diluted commercial antibodies against RBD (GenScript Cat#A02038) or N (Acrobiosystems Cat#NUN-S41) starting from a 1 mg/mL concentration. Negative control samples were prepared with PBS. After a 30 minute incubation (RT protected from light), samples were washed three times in PBS. Secondary antibodies were diluted in 100 µL of PBS containing 5% FBS: anti-human IgG-PE (1:50) (Clone G18-145, BD Biosciences Cat#555787) and anti-human IgM-BV421 antibodies (1:1000) (Clone G20-127, BD Biosciences Cat#555783). The mix was incubated with the samples for 15 min at RT protected from light. One final wash was performed, and microbeads were resuspended in 200 µL of PBS supplemented with 5% FBS for acquisition. At least 600 events for each type of

	microbead were acquired in a FACSymphony flow cytometer (BD Biosciences) and geometric mean fluorescence intensities (gMFI) were obtained. Results were analyzed using FlowJo version 10 (BD Biosciences).
Instrument	FACSymphony flow cytometer (BD Biosciences).
Software	Data was collected and analyzed using FACSDiva version 8.0.2 and FlowJo version 10 (BD Biosciences), respectively.
Cell population abundance	A minimum of 600 events were collected for each bead population.
Gating strategy	Positive events were determined based on the signal of the uncoated beads.

☒ Tick this box to confirm that a figure exemplifying the gating strategy is provided in the Supplementary Information.
